# Factors associated with non-home discharge of patients hospitalized for hip fracture: A nationwide retrospective study using the Japanese diagnostic procedure combination database

**DOI:** 10.1097/MD.0000000000033138

**Published:** 2023-03-03

**Authors:** Mutsuko Moriwaki, Kenshi Hayashida, Yasuko Ogata

**Affiliations:** a Quality Management Center, Tokyo Medical and Dental University Hospital (TMDU), Bunkyo-ku, Tokyo, Japan; b Department of Medical Informatics and Management, University Hospital, University of Occupational and Environmental Health, Kitakyushu, Fukuoka, Japan; c Graduate School of Health Care Sciences, Tokyo Medical and Dental University (TMDU), Bunkyo-ku, Tokyo, Japan.

**Keywords:** administrative data, elderly, hip fracture, Japan, nonhome discharge, patients

## Abstract

In Japan, the length of stay in acute care hospitals has been shortened, home medical care has been promoted following national policy. However, many issues remain in promoting home medical care. The aim of this study was to clarify the profiles of patients with hip fractures, aged ≥ 65 years, who were hospitalized in acute care institutions at the time of discharge and the influence on nonhome discharge. This study used data from patients who satisfied all the following conditions: Patients aged ≥ 65 years who were hospitalized and discharged between April 2018 and March 2019; Patients with hip fractures, and; Patients who were admitted from home. The patients were classified into the home discharge and nonhome discharge groups. Multivariate analysis was conducted by comparing socio-demographic status, patient background factors, patient status at discharge, and hospital function. This study included 31,752 patients (73.7%) and 11,312 patients (26.3%) in the nonhome discharge group and home discharge group, respectively. Overall, the proportions of males and females were 22.2% and 77.8%, respectively. The average (standard deviation) age of the patients was 84.1 years (7.4) and 81.3 years (8.5) in the nonhome discharge and home discharge groups, respectively (*P* < .01). The following factors affected nonhome discharge: 75 to 84 years (odds ratio [OR] = 1.81, 95% confidence interval [CI] = 1.68–1.96), ≥85 years (OR = 2.17, 95% CI = 2.01–2.36), electrocardiography or respiratory treatment “(Factor A3) (OR = 1.44, 95% CI = 1.23–1.68), level of assistance with activities of daily living “(Factor B1)” (OR = 4.56, 95% CI = 4.22–4.92), and hospital where the patient-to-nurse ratio is 7:1 (OR = 2.12, 95% CI = 1.91–2.35). The results suggested that support from activities of daily living caregivers and implementing medical treatments such as respiratory care are required to advance home medical care. This study’s method enables analysis focusing on aspiration pneumonia and cerebral infarction, which are common among older adults. Furthermore, specific measures for promoting home medical care for patients who are highly dependent on medical and long-term care may be developed.

## 1. Introduction

The number of hospital beds per capita in Japan is higher than that in Western countries, and the length of hospital stay is longer, indicating the overprovision of medical care.^[1,2]^ Additionally, as medical expenses increase, the appropriateness of medical care is being incorporated into national policy and functional differentiation of medical care is rapidly progressing. In Japan, the length of stay in acute care hospitals has been shortened, home medical care has been promoted in accordance with national policy. However, many issues remain in promoting home medical care. Owing to an increase in the number of patients who are highly dependent on medical care, an emerging issue is the home medical care system, and with family members and caregiver support as the core of this system. Hip fractures, which consist of femur and femoral neck fractures, are traumatic injuries that are likely to occur among older adults. Additionally, such injuries will probably become more common in the future as the population ages. There are many studies on discharge destinations after the treatment of patients with hip fractures, including in the West.^[3–6]^ However, few such studies exist in Japan, the number of analyzed participants is small.^[7–9]^ Many injured patients cannot return to their daily life preinjury^[5,10]^ and cannot return to their home before the onset of the injury^[11]^ owing to the large invasiveness on physical function. Moreover, over half of injured patients live at home after receiving acute medical care following hospitalization.^[3,12–14]^

Discharge destinations of older adults largely depend on the patient’s physical status,^[3,15]^ insurance system, medical policy, housing environment,^[16]^ and socio-economic impact; thus, differences between countries emerge.^[17–19]^ Clarifying the issues in Japan by comparing factors that make it difficult for elderly patients with hip fractures to return home with previous overseas research is necessary to promote home medical care.

Therefore, the aim of this study was to clarify the profiles of patients with hip fractures, aged ≥ 65 years, who were hospitalized in acute care institutions at the time of discharge and the impact on nonhome discharge.

## 2. Methods

### 2.1. Data source

This retrospective study utilized the diagnosis procedure combination (DPC) and severity of a patient’s condition and the extent of a patient’s need for medical/nursing care (SCNMN) databases.

The DPC is a patient classification method for acute inpatients developed in Japan as a tool to make acute medical care transparent and visible. In 2003, the Ministry of Health, Labour, Welfare implemented this as a lump-sum per-diem payment system, and it is used for acute inpatient medical care and medical resource allocation. Acute care hospitals in Japan are part of this system and report medical information on medical procedures to the Ministry of Health, Labour, and Welfare.^[20,21]^ As of 2020, the DPC database had been applied to 1757 facilities and 483,180 beds, accounting for 24.5% of Japan’s general hospitals and 54.4% of its beds. This DPC database collects the following information: patient age and sex; main diagnoses, preexisting comorbidities, postadmission complications linked with the international classification of diseases, and 10^th^ revision codes; dates of admission and discharge; route of hospital admission; discharge destination; discharge outcome; and surgical procedure.^[20,22]^

The SCNMN database is an index developed in Japan for measuring the nursing services required by inpatients. It is now mainly used as a standard for paying medical expenses such as basic hospital charges for acute care. This index consists of 21 items divided into 3 categories. Item A (7 items) refers to highly specialized nursing care, including monitoring and treatment. Item B (7 items) refers to patients’ functional status, such as activities of daily living (ADL), which influence medical care. Item C (7 items) refers to medical management, such as surgical treatment and emergency care. These items are evaluated daily for each patient and compiled into a database.^[23]^

### 2.2. Study population

Inclusion criteria were: Patients aged ≥ 65 years who were hospitalized and discharged between April 2018 and March 2019; Patients with hip fractures (DPC code; 160800xx01xxxx); and; Patients who were admitted from home.

Exclusion criteria were: Patients who died after discharge; Patients with missing values in SCNMN items A and B; and; Patients with outlier age or length of stay values.

### 2.3. Outcomes

The outcome was “nonhome discharge.” DPC database collects discharge destination information. Among this information, we defined patients who entered the information of “transfer to medical institution” and “nursing facility or welfare facility” as “nonhome discharge.”

### 2.4. Variables

We utilized variables that influence nonhome discharge based on previous research.^[3–9]^ The variables related to socio-demographic status were sex and age, and those related to patient background factors included the presence or absence of dementia, comorbidities, presence or absence of home medical care before hospital admission, and length of stay. Comorbidities were calculated using the Charlson comorbidity index^[24]^ and classified using 1, 2, or ≥ 3 points. Variables associated with patient status at discharge used SCNMN items A and B (Additional File 1). The following facility factors were established: designated city by government ordinance, hospital bed scale, and hospital with a patient-to-nurse ratio of 7:1.^[25]^ Hospitals in the DPC system have a patient-to-nurse ratio of either 7:1 or 10:1 in general wards.

### 2.5. Statistical analyses

We conducted statistical analyses using the Kolmogorov–Smirnov test and according to the following procedure. We compared the socio-demographic status, patient background factors, and patient status at the time of discharge between the groups based on whether it was a home or nonhome discharge (Chi-squared or Mann–Whitney *U* test).

Variables relating to patient status at discharge include items with a mutual impact; thus, factor analysis was conducted for each SCNMN items A and B (promax rotation). Four factors were extracted out of the 9 items for SCNMN Item A (Table S1, Supplemental Digital Content, http://links.lww.com/MD/I573, which illustrates the procedure for aggregating patient status assessments from SCNMN item A). These 4 factors and 6 items with factor loadings < 0.3 were extracted as variables related to SCNMN Item A. Factor analysis was similarly conducted for SCNMN Item B, and 2 factors were extracted from 6 items (Table S2, Supplemental Digital Content, http://links.lww.com/MD/I574, which illustrates the procedures for aggregating patient’s level of assistance with ADLs from SCNMN item B). The second factor of SCNMN Item B, the variable of “Engaged in dangerous behavior,” was merged with the variable of dementia. Subsequently, we selected 1 or the other and determined the final variables for variables that exhibited a high correlation between items among those where comparisons between groups showed a significant difference and those that were extracted from factor analysis. We then conducted a logistic regression analysis with nonhome discharge as the dependent variable. IBM SPSS.28 was used for statistical analysis.

Although sample size calculations were not conducted, the effect size was calculated using a post hoc power analysis.

## 3. Results

**Figure 1.**
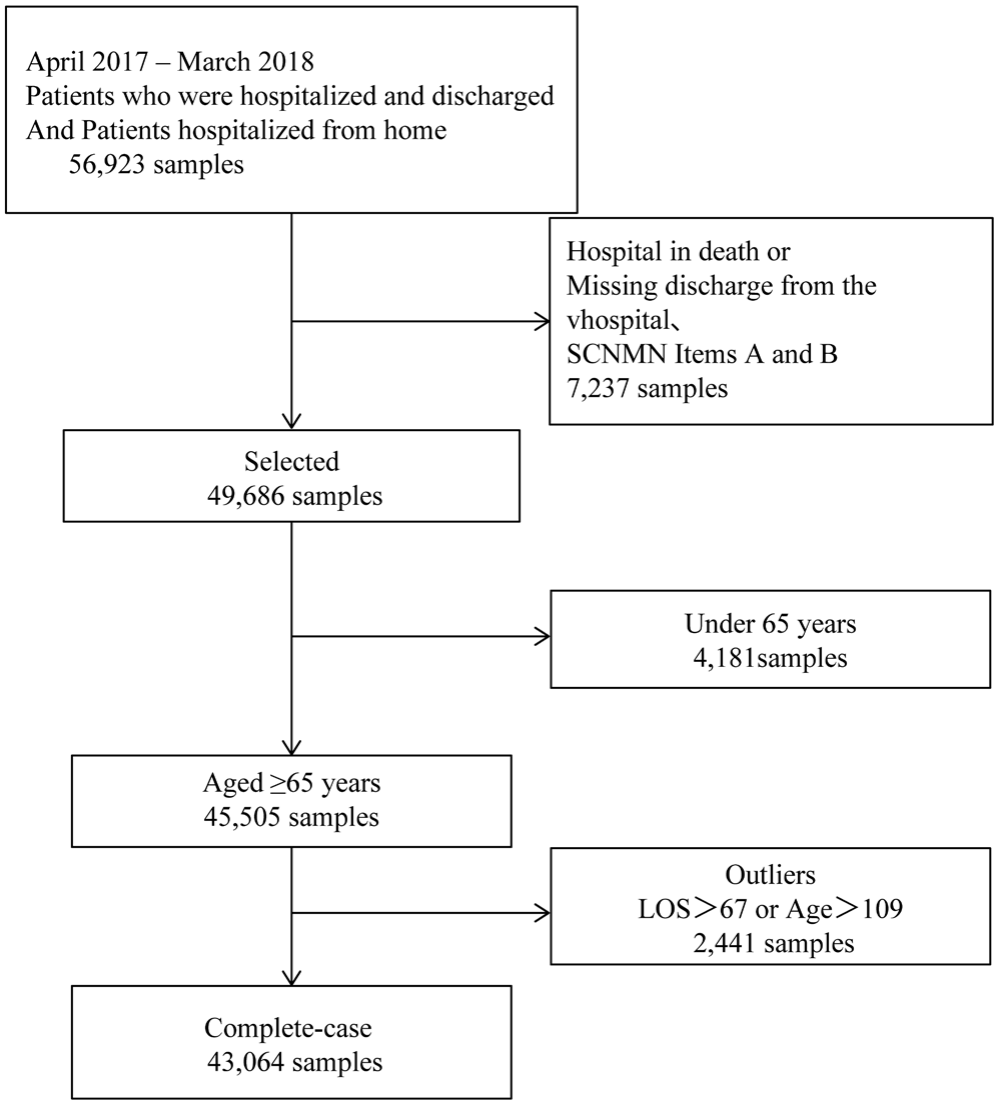
Flowchart of patient selection. Patients aged > 65 years and who were hospitalized and discharged were included. Patients were excluded if they died in the hospital, had missing data (discharge from the hospital and SCNMN items A and B), or were an outlier (LOS or age). Abbreviations: LOS, Length of stay; SCNMN, severity of a patient’s condition and the extent of a patient’s need for medical/nursing care.

The number of patients with hip fractures hospitalized from home during the analysis period was 56,923; after excluding cases of death, those under 65 years, and outlier age and length of stay values, we analyzed 43,064 cases (Fig. 1).

The study included 31,752 patients (73.7%) and 11,312 patients (26.3%) in the nonhome discharge group and home discharge group, respectively. In the nonhome discharge and home discharge groups, the average (standard deviation [SD]) age of the patients was 84.1 years (7.4) and 81.3 years (8.5) years, respectively (*P* < .01); the number of patients with dementia (%) was 5808 (18.3) and 1721 (15.2), respectively (*P* < .01); and the number of patients with home medical care before admission (%) was 1582 (5.0) and 577 (5.1), respectively (*P* = .65) (Table 1). The number of patients in medical institutions with a patient-to-nurse ratio of 7:1 (%) was 25,760 (81.1%) and 8507 (75.2) in the nonhome discharge group and home discharge group, respectively (*P* < .01). The nonhome discharge group had a larger number of patients in medical institutions with at least 400 beds (*P* ≤ .01). For the patient status at discharge, the following 8 items were significantly different between the nonhome discharge group and home discharge group for SCNMN Item A: “wound treatment (excluding pressure ulcer treatment),” “pressure ulcer treatment,” “respiratory care (except for only sputum aspiration),” “management of 3 or more intravenous lines simultaneously,” “electrocardiography (ECG) monitor management,” “syringe driver management,” “internal use of narcotics, application, management of suppositories,” and “use of continuous infusion of antithrombotic embolic drugs.” For SCNMN Item B, the following factors had a higher percentage in the nonhome discharge group: patients requiring partial or full assistance for items relating to ADL, patients unable to receive medical care and treatment directions, and patients engaged in dangerous behavior (Table 2).

**Table 1:** Patient characteristics.

	Nonhome discharge group		Home discharge group		*P* value	Effect size
	n = 31,752		n = 11,312			
Age, mean, SD	84.1	7.4	81.3	8.5	.00	0.37
Sex, n, %					.04	0.01
Male	6967	21.9	2587.0	22.9		
Female	24,785	78.1	8725.0	77.1		
Age class, n,%					.00	0.17
65–74	3667	11.5	2729	24.1		
75–84	11,552	36.4	4243	37.5		
≥85	16,533	52.1	4340	38.4		
Dementia, n, %	5808	18.3	1721	15.2	.00	0.04
CCI score, n, %					.00	0.15
0 point	13,652	43.0	5139	45.4		
1 point	10,154	32.0	3310	29.3		
2 points	4783	15.1	1716	15.2		
≥3 points	3163	10.0	1147	10.1		
Home medical care before hospitalization	1582	5.0	577	5.1	.65	0.02
Length of stay, mean, SD	26.31	11.457	33.70	14.460	.00	-0.59
Urban	8401	26.5	2650	23.4	.00	0.02
7:1 patient-to-nurse ratio	25,760	81.1	8507	75.2	.00	0.15
Bed size					.00	0.18
<200	2288	7.2	1862	16.5		
200–399	9593	30.2	4157	36.7		
400–599	9648	30.4	2589	22.9		
≥600	5674	17.9	1311	11.6		

p: continuous variable; Mann-Whitney *U* test, discrete variable; χ2 test. Effect size: Mann–Whitney *U* test; Cohen d, χ2 test; Cramer V.

CCI = Charlson comorbidity index, SD = standard deviation.

**Table 2:** Patient condition at discharge (SCNMN Item A and Item B).

	nonhome discharge group		Home discharge group		*P* value
	n = 31,752		n = 11,312		
SCNMN item A					
Wound treatment (excluding treatment of pressure ulcer)	1521	4.8	307	2.7	.00
Treatment of pressure ulcer	56	0.2	10	0.1	.04
Respiratory care (except for only sputum aspiration)	576	1.8	106	0.9	.00
Management of 3 or more intravenous lines at the same time	96	0.3	12	0.1	.00
ECG monitor management	1049	3.3	196	1.7	.00
Syringe driver management	26	0.1	2	0.0	.02
Management of blood transfusion and blood product	57	0.2	12	0.1	.09
Use of antineoplastic agents (injection only)	13	0.0	5	0.0	.88
Management of oral administration of antineoplastic agents	206	0.6	83	0.7	.34
Use of narcotics (injection only)	8	0.0	3	0.0	.94
Internal use of narcotics, application, management of suppositories	39	0.1	25	0.2	.02
Radiation therapy	0	0.0	3	0.0	.00
Immunosuppressant management	647	2.0	239	2.1	.63
Use of pressor agent (injection only)	32	0.1	6	0.1	.14
Use of antiarrhythmic agent (injection only)	7	0.0	0	0.0	.11
Use of continuous infusion of antithrombotic embolic drug	58	0.2	10	0.1	.03
Drainage management	24	0.1	4	0.0	.15
Treatment in a sterile treatment room	0	0.0	0	0.0	
SCNMN item B					
Turnover					.00
Yes	5374	16.9	3423	30.3	
Can do if grabbing something	17,354	54.7	3790	33.5	
No	7558	23.8	1290	11.4	
Transfer					.00
Without assistance	6682	21.0	4624	40.9	
Partly assisted	19,247	60.6	3061	27.1	
Fully assisted	4357	13.7	818	7.2	
Oral care, with assistance	24,127	76.0	4051	35.8	.00
Meal intake					.00
Without assistance	11,381	35.8	5336	47.2	
Partly assisted	17,084	53.8	2679	23.7	
Fully assisted	1821	5.7	488	4.3	
Personal dressing					.00
Without assistance	6662	21.0	4417	39.0	
Partly assisted	11,129	35.0	1927	17.0	
Fully assisted	12,495	39.4	2159	19.1	
Able to receive directions on medical care and treatment	8975	28.3	2025	17.9	.00
Engaged in dangerous behavior	5493	17.3	1205	10.7	.00

ECG = electrocardiography, SCNMN = severity of a patient’s condition and the extent of a patient’s need for medical/nursing care.

Regarding factors that affected nonhome discharge, the following had a positive influence: 75 to 84 years (odds ratio [OR] = 1.81, *P* < .01), ≥85 years (OR = 2.17, *P* < .01), ECG or respiratory treatment (Factor A3) (OR = 1.44, *P* < .01), level of assistance with ADLs (Factor B1) (OR = 4.56, *P* < .01), and hospital with a patient-to-nurse ratio of 7:1 (OR = 2.12, *P* < .01); and the following had a negative influence: dementia or dangerous behavior (Factor B2) (OR = 0.91, *P* < .01). The length of stay had almost no effect (OR = 0.97, *P* < .01) (Table 3).

**Table 3:** Logistic regression analysis of the effect of nonhome discharge.

	B	Exp (B)	95% CI		*P* value
			Lower	Upper	
Patient background					
Age class					
65–74 (reference)					.00
75–84	0.60	1.81	1.68	1.96	.00
≥85	0.78	2.17	2.01	2.36	.00
CCI score (excluding dementia)					
0 point (reference)					.48
1 point	0.03	1.03	0.97	1.10	.36
2 points	−0.04	0.96	0.88	1.04	.33
≥3 points	0.00	1.00	0.90	1.10	.94
Length of stay	−0.03	0.97	0.97	0.98	.00
Patients condition					
Wound treatment (excluding treatment of pressure ulcer)	0.00	1.00	0.87	1.16	.95
Treatment of pressure ulcer	0.49	1.63	0.73	3.66	.24
Internal use of narcotics, application, management of suppositories	−0.74	0.48	0.26	0.89	.02
Use of continuous infusion of antithrombotic embolic drug	0.27	1.31	0.63	2.72	.47
Factor A1 (blood transfusion/intravenous lines)	0.27	1.30	0.72	2.35	.38
Factor A2 (pressor agent/narcotics/Syringe)	0.35	1.42	0.65	3.08	.38
Factor A3 (ECG/respiratory)	0.36	1.44	1.23	1.68	.00
Factor B1 (Level of assistance with ADLs)	1.52	4.56	4.22	4.92	.00
Dementia Or factor B2 (directions/dangerous behavior)	−0.10	0.91	0.85	0.97	.00
Hospital Factor					
7:1 patient-to-nurse ratio	0.75	2.12	1.91	2.35	.00
Urban	0.03	1.04	0.97	1.10	.28

Hosmer-Lemeshow < 0.01. Nagelkerke R2 = 0.15. Dependent variable:nonhome discharge = 1.

ADLs = activities of daily living, CI = confidence interval, ECG = electrocardiography.

## 4. Discussion

In this study, we used a large-scale database of acute care hospitals to clarify the profiles of patients with hip fractures in Japan at the time of discharge that influence whether their discharge destination is hospital transfer or facility (nonhome).

Factors for nonhome discharge include advanced age and decreased ADL levels,^[3,11,15,17–19]^ and the same results were obtained in this study. In this study, the nonhome discharge group had a significantly longer length of stay (7 days longer) than the home discharge group, but the multivariate analysis showed no effect (OR = 0.97, *P* < .01). This is expected to affect Japan’s medical and long-term care (LTC) policies. In Japan, many acute care hospitals are enrolled in the DPC system,^[20]^ which is based on comprehensive payments. Under this system, the amount of medical fees per day will be reduced as the hospitalization period is extended, starting from hospitalization period II (period based on the median hospitalization period for each DPC). Therefore, discharge adjustments are conducted considering the length of hospital stay from a financial management perspective. Hospitalization period II for the current injury is set as a length of stay between 13 and 24 days (hospitalization period I (up to 12th day): 25,210 yen/day; II (up to 24th day): 18,630 yen/day; III (up to 60th day): 15,840 yen/day). Additionally, medical institutions with a “7:1 patient-to-nurse ratio” could influence nonhome discharge (OR = 2.12, *P* < .01) because they may be receiving medical fee incentives. Each medical institution in Japan pays different levels of basic hospitalization fees, and each level has different facility standards. Patient-to-nurse ratios are also stipulated in these standards.^[25]^ The 7:1 patient-to-nurse ratio is the highest level among the basic hospitalization fees for general acute care (excluding severe beds such as the intensive care unit or high care). It is desirable for medical institutions that fall under this basic hospitalization fee to transfer patients to a logistical support hospital as soon as possible after completing acute care because of medical fees. A Dutch study reported that the length of stay of patients with hip fractures is as follows: 8.5 days (SD 6.7) for all patients, 6.5 days (SD 6.1) for the “Discharge home” group, and 10.8 days (SD 8.8) for the “Nursing home” group.^[15]^ Furthermore, a study in the United States reported that the length of stay of patients with hip fractures is 6.68 days.^[26]^ Progress has been made on policies to reduce the length of stay in Japan; however, the length of stay in Japan is more than 3 times longer than that reported in these studies. Acute care hospitals in Japan could serve as facilities that include the functions of acute care hospitals in other countries and those of geriatric rehabilitation centers and nursing homes with regard to this injury.

An extended length of hospital stay has been associated with serious patient status.^[27–33]^ In Japan, the deterioration of patients general condition might influence the extension of the length of stay.^[34]^ However, the following reasons were considered for the extension of the length of hospital stay in the home discharge group compared with the nonhome discharge group. The hip fracture studied was a traumatic injury, given its characteristics, the home discharge group may be patients who require assistance, such as improving ADL, and patients whose daily life is independent but may require assistance such as being watched over in their daily activities. In Japan, LTC insurance was introduced in 2000 as a mechanism to support the care of the elderly in society.^[35]^ Therefore, comprehensive healthcare and welfare services are now available, which have greatly promoted home care. However, it is generally stated that receiving LTC services takes over a month from the time of application. Moreover, those who are not covered by LTC services will need to introduce the service at their own expense if they require assistance in their daily lives. The results of this study, which showed that the home discharge group had a longer hospital stay than the nonhome discharge group despite favorable ADL levels, may have been influenced by such LTC procedures. The transition from inpatient care to home care signifies a shift from health care insurance to LTC insurance, and these results suggest the procedural issues in this shift.

The results of this study, which indicated that requiring ADL support is likely to lead to nonhome discharge, were consistent with those of previous studies.^[3,7,15]^ Over 70% of the participants in this study were unable to return home after discharge. Tanaka et al^[7]^ reported that, in their target hospital, a higher percentage of patients aged 80 years or younger who underwent hip surgery was discharged to homes or nursing homes in 2011 to 2013 than in 2001 to 2003. This was a single-center study; however, the results suggest that there was progress in hospital function differentiation and home medical care based on national policy. Furthermore, regarding subsequent discharge destinations of patients who were admitted to rehabilitation facilities after being discharged from hospitals, it was reported that patients discharged to home had a significantly higher Barthel Index than patients discharged to nursing homes, indicating the possibility of patients being able to return home if ADL increases.

The need for ECG monitoring and respiratory care may influence home discharge. This might reflect the difficulty in acquiring skills and the strong sense of resistance to home management. Medical treatment in home medical care imposes heavy burdens on caregivers and it has been shown that home-visit nursing care and short stays are effective in reducing this burden.^[36,37]^

In Japan, the annual number of new patients with hip fractures might reach 240,000 in 2020 and 320,000 in 2040 owing to an aging society.^[38]^ Home medical care and LTC are advancing with the introduction of LTC insurance, but Japan continues to rely on hospital support more than other countries.^[15,28]^ Therefore, further system development is required to support daily life, including ADL assistance in home medical care, support for the medical treatment implementation, and a seamless transition from medical insurance to LTC insurance.

This study has some limitations. This was conducted using the DPC database; hence, the results are dependent on information obtainable from the database. The DPC database contains clinical process information based on the daily allowance lump-sum system for acute care facilities in Japan. The patient discharge destination is also influenced by the family background of the caregiver (including the presence or absence of family members living together), financial situation of family members, number of medical/nursing facilities, and local medical provision systems such as the amount of services. However, these aspects have not been investigated given database limitations. In addition, medical institutions that are not covered by the lump-sum daily allowance system also accept patients with hip fractures. If medical institutions accepting “Hip fracture” are taken as the population of this study, medical institutions not covered by the daily allowance lump-sum system are not included. Therefore, hospital factors may not be generalizable. From this study, the condition of patients discharged to nonhome destinations was clarified from the perspective of patients discharged from acute care hospitals. Following discharge from a medical institution, patients needs for home medical care need to be analyzed, taking into consideration the fact that the patient may return home through various medical or nursing facilities such as rehabilitation facilities or nursing homes. The ultimate research goal should be to demonstrate that home care leads to a better quality of life and longer life expectancy. As the present study did not show this, further research investigating whether home care leads to better outcomes for the elderly is needed.

In conclusion, this study clarified that ADL, age, medical procedure status, and hospital factors affected whether a patient would be discharged to a destination other than their home. This is the reason for promoting home medical care in Japan. This is the only study that utilizes data from multiple centers to identify factors in patients unable to be discharged to their home. Using this method to analyze diseases common among the elderly may lead to specific measures to promote home care for patients who are highly dependent on medical and nursing care.

## Acknowledgment

The authors thank Shunji Shimoda (Department of Clinical Data Management and Research, Clinical Research Center) of the National Hospital Organization Headquarters for their technical support in the extraction and management of patient data.

## Author contributions

**Conceptualization:** Kenshi Hayashida.

**Data curation:** Mutsuko Moriwaki.

**Formal analysis:** Mutsuko Moriwaki.

**Funding acquisition:** Kenshi Hayashida.

**Investigation:** Mutsuko Moriwaki, Kenshi Hayashida.

**Methodology:** Mutsuko Moriwaki, Kenshi Hayashida.

**Project administration:** Mutsuko Moriwaki, Yasuko Ogata.

**Resources:** Mutsuko Moriwaki, Kenshi Hayashida.

**Software:** Mutsuko Moriwaki.

**Supervision:** Yasuko Ogata.

**Validation:** Yasuko Ogata.

**Visualization:** Mutsuko Moriwaki.

**Writing – original draft:** Mutsuko Moriwaki.

**Writing – review & editing:** Kenshi Hayashida, Yasuko Ogata.

## Supplementary Material




