# Supplementary material for: Factors associated with non-home discharge of patients hospitalized for hip fracture: A nationwide retrospective study using the Japanese diagnostic procedure combination database
Source: Medicine (Baltimore). 2023 Mar 3;102(9):e33138. doi: 10.1097/MD.0000000000033138 (PMC9981375; doi:10.1097/MD.0000000000033138)
Supplement: Supplementary file 1 [file medi-102-e33138-s001.pdf]

Supplemental Table 1 Factor analysis for highly specialized nursing care including monitoring and treatment (Item A)

	Factor A1 (blood transfusion/intravenous lines)	Factor A2 (pressor agent/narcotics/Syringe)	Factor A3 (ECG/respiratory)	Factor A4 oral administration of antineoplastic agents
Management of blood transfusion and blood product	0.86	-0.05	0.00	0.15
Management of 3 or more intravenous lines at the same time	0.82	0.06	0.00	-0.13
Use of pressor agent (injection only)	0.00	0.53	0.03	0.16
Use of narcotics (injection only)	-0.01	0.51	-0.04	0.04
Syringe driver management	0.06	0.26	0.02	-0.15
ECG monitor management	0.00	-0.02	0.49	0.00
Respiratory care (except only sputum aspiration)	0.00	0.02	0.43	-0.03
Management of oral administration of antineoplastic agents	0.00	0.00	0.00	-0.01

※1 promax rotation

※2 A factor analysis was performed to aggregate the patient status assessments from SCNMN item A and use them as variables in multivariate analyses. The items of item A used in the factor analysis are those with a significant difference in the comparison between the two groups as shown in T2.

Abbreviation: ECG, electrocardiography; SCNMN, severity of a patient's condition and the extent of a patient's need for medical/nursing care
