# Supplementary material for: Factors associated with non-home discharge of patients hospitalized for hip fracture: A nationwide retrospective study using the Japanese diagnostic procedure combination database
Source: Medicine (Baltimore). 2023 Mar 3;102(9):e33138. doi: 10.1097/MD.0000000000033138 (PMC9981375; doi:10.1097/MD.0000000000033138)
Supplement: Supplementary file 2 [file medi-102-e33138-s002.pdf]

Supplemental Table 2 Factor analysis for patients' functional status such as activities of daily living

(Item B)

	**Factor B1 (level of assistance with ADLs)**	**Factor B2 (directions/dangerous behavior)**
Transfer	0.75	-0.03
Oral care	0.79	-0.04
Meal intake	0.69	0.08
Personal dressing	0.80	0.01
Able to receive directions on medical care and treatment	0.06	0.80
Engaged in dangerous behavior	-0.06	0.70

※1 Principal Factor Method

※2 A factor analysis was performed to aggregate the patient's level of assistance with activities of daily living from SCNMN item B and use them as variables in multivariate analyses. The items of item B used in the factor analysis are those with a significant difference in the comparison between the two groups as shown in T2.

Abbreviations: ADLs, activities of daily living; SCNMN, severity of a patient's condition and the extent of a patient's need for medical/nursing care
